# Psychometric Properties of the Attitudes toward Self-Revised in Italian Young Adults

**DOI:** 10.1155/2013/209216

**Published:** 2013-06-26

**Authors:** Marco Innamorati, Stella Tamburello, Anna Contardi, Claudio Imperatori, Antonino Tamburello, Aristide Saggino, Michela Balsamo

**Affiliations:** ^1^Università Europea di Roma, 00163 Rome, Italy; ^2^Istituto Skinner, 00184 Rome, Italy; ^3^DISPUTer, Dipartimento di Scienze Psicologiche, Umanistiche e del Territorio, “G. d'Annunzio” University, 66100 Chieti, Italy

## Abstract

*Objectives and Methods*. Several researchers have provided support for the critical role of cognitive vulnerabilities in the development of depression. The Attitudes toward Self-Revised (ATS-R) was designed to assess three potential self-regulatory vulnerabilities to depression: High Standards (HS), Self-Criticism (SC), and Negative Generalization (NG). The aim of the study was to assess the psychometric properties of the ATS-R in the Italian young adult population. The ATS-R, the Beck Depression Inventory-II (BDI-II), the Beck Hopelessness Scale (BHS), and the Teate Depression Inventory (TDI) were administered to 857 (320 men and 537 women) young adults. *Results*. The best-fitting solution for the ATS-R was a 2-factor model, which obtained satisfactory homogeneity of content (HS/SC: Cronbach *α* = 0.81; mean interitem correlation = 0.46. NG: Cronbach *α* = 0.75; mean interitem correlation = 0.43) and significant correlation with the BDI-II (NG: Pearson *r* = 0.29, *P* < 0.01), the TDI (HS/SC: Pearson *r* = −0.26, *P* < 0.01), and the BHS (HS/SC: Pearson *r* = −0.29, *P* < 0.01; NG: Pearson *r* = 0.22, *P* < 0.01). *Conclusions*. The Italian version of the ATS-R seems to be a valid instrument for the study of the role of cognitive tendencies as potential vulnerability for depression.

## 1. Introduction

Depression involves a wide variety of pathological conditions which vary along a continuum from more mild to more severe and persistent forms, such as major depressive disorder (MDD). MDD is the most widespread psychiatric disorder in the world [1–4], although its prevalence differs between countries around the world [5].

At the end of the eighties, the Epidemiological Catchment Area (ECA) study investigated the prevalence of MDD in the US general population and reported 30-day prevalence rates ranging between 1.7% and 3.4% [6]. More recently, the National Comorbidity Survey Replication (NCS-R) estimated a 12-month prevalence of 6.6% [7]. In Italy, the European study on the Epidemiology of Mental Disorders (ESEMeD) estimated rates of 3.0% and 10.0%, respectively, for the 12-month and lifetime MDD prevalence [8].

MDD is one of the major causes of disability, currently estimated as the fourth cause for the global burden of diseases [9, 10], and expected to become the second one within the year 2020 [9].

Numerous etiological models have been proposed and studied to explain how individuals become depressed. Cognitive theories of depression postulate that the dysfunctional interpretation of a life event may lead the individual to be vulnerable to depression following the occurrence of the stressful event. The most renowned cognitive theories of depression are the hopelessness theory [11] and the Beck's theory [12, 13]. Abramson et al. [11] focused on three maladaptive depressogenic inferential styles which predispose individuals to the development of depression: (1) the tendency to attribute negative events to global and stable causes; (2) the tendency to perceive negative events as having many disastrous consequences; and (3) the tendency to view the self as flawed or deficient following negative events. Beck [12, 13] hypothesized that depressed people have unrealistical and dysfunctional beliefs about the self, the world, and the future, a set of faulty cognitive processes known as the “cognitive triad.”

Several researches have provided support for the critical role of cognitive vulnerability (i.e., a trait-like tendency to interpret information in negative and distorted ways in face of subjectively perceived stressful events [14]) in the development of depressive disorders [15–18]. For example, Evans et al. [18] found that holding a negative self-schema is an independent risk factor for the onset of depression in women, whereas Alloy et al. [16] reported that a negative inferential style increases the risk for the initial onset and recurrence of depressive episodes.

According to Carver and Ganellen [19], self-punitiveness is a salient feature of depression, associated with the holding of overly high standards, the tendency to be too critical with the self for failing to attain a standard, and the tendency to generalize from a single failure to the broader sense of self-worth. In order to confirm their hypothesis and to assess the relationship among these three processes and depression, the authors developed the Attitudes toward Self (ATS). The ATS was composed of 18 items divided into three subscales: Negative Generalization, High Standards, and Self-Criticism. In 1988, the authors elaborated a revised version of the measure (ATS-R) with a reduced number of items, focusing more explicitly on the cognitive tendencies than the original items [20]. Several studies indicated that, among the three tendencies, negative generalization was the only one reliably related to depression and mediating the relationship between life events and depression [20, 21] and between negative events and self-esteem [22]. Studies constantly indicated that homogeneity of content of the three factors was sufficient (Cronbach alpha > 0.70), except for self-criticism [20–24]. 

This analysis of the literature suggests the importance to assess cognitive vulnerabilities (CV) to depression and the utility of the ATS-R in measuring three tendencies potentially associated with a higher risk to develop depression. Thus, the aim of the study was to assess the psychometric properties of the ATS-R [20] in the Italian young adult population.

## 2. Materials and Methods

### 2.1. Participants

Participants were 857 (320 men and 537 women) Italian young adults recruited between January 2011 and May 2011 in Central Italy. Mean age of the sample was 22.41 years (SD = 4.36; range: 18–34 years). Inclusion criteria included age between 18 and 34 years and the ability to read and write in Italian. Exclusion criteria included the presence of any condition affecting the ability to complete the assessment, including illiteracy and denial of informed consent.

The sample was nonrandomly recruited through attendants of adult education classes and advertisement posted to established community groups. More than 89% of the respondents had completed high school at the time of the assessment, and 55.5% of them were university students. Educational attainment in our sample was higher than the national average (according to the National Institute of Statistics (http://www.istat.it/) in Central Italy around 80% of people 20–24 years old complete secondary school, and 40% of people 19–25 years old attend university), and our sample should not be considered representative of the Italian young adult population.

All participants took part voluntarily in the study, gave informed consent, and completed the assessment anonymously.

### 2.2. Measures

All participants were administered the ATS-R, the BDI-II, the Teate Depression Inventory (TDI) [25], and the Beck Hopelessness Scale (BHS) [26]. The assessment protocol was self-administered; the questionnaires were delivered to participants in a group setting or individually and completed in the presence of a researcher, who possibly helped with the compilation.

The ATS-R [20] is a 10-item scale measuring three potential self-regulatory vulnerabilities to depression (holding overly high standards, the tendency to be self-critical at any failure to perform well, and the tendency to generalize from a single failure to the broader sense of self-worth). Compared to the previous version [27] ATS-R is characterized by new items focused more explicitly on the cognitive tendencies than the originals, and its shortness reflects the straightforwardness of each cognitive tendency [21, 23].

Respondents are asked to rate the degree to which they agree/disagree with each statement on a 5-point Likert-type scale (ranging from “*I agree a lot*” to “*I disagree a lot*”). The Italian version of the ATS-R was translated from the original English version into Italian by two authors of the present study (Marco Innamorati and Michela Balsamo); then, the Italian version was independently and blindly back-translated by a native English speaking researcher. 

The BDI-II is a well-known self-report inventory composed of 21 items designed to assess the presence and severity of depressive symptoms, according to DSM-IV [28] criteria. Respondents endorse specific statements reflecting their feelings over the last two weeks, including today. Each statement is rated on a 4-point Likert-type scale ranging from 0 to 3, based on the severity of depressive symptoms. Importantly, the extensive literature has supported the psychometric properties of the scale in clinical and nonclinical samples [29, 30]. In the current study, Cronbach alpha index was 0.87.

The BHS is a 20-item scale for measuring negative attitudes about the future [26]. When responding to the 20 true-false items on the BHS, individuals either endorse a pessimistic statement or deny an optimistic statement. Research has consistently supported a positive significant relationship between BHS scores and measures of depression, suicidal intent, and suicidal ideation [31–35]. Studies on the Italian population have been carried out successfully [36] and have led to validation of the scale [37]. In the present study, Cronbach alpha was 0.84.

The TDI is a new 21-item self-report instrument designed to assess the *Diagnostic and Statistical Manual of Mental Disorders*, fourth edition, text revision (DSM-IV-TR) [28], criteria for major depression [25]. It was developed via Rasch logistic analysis of responses [38, 39], in order to overcome psychometric inherent weaknesses of existing measures of depression [30]. Each item is rated on a 5-point Likert-type scale ranging from 0 (always) to 4 (never). The total score ranges from 0 to 84, with higher scores indicating more severe depressive symptoms. In the standardization samples, the TDI displayed very good psychometric properties (with an excellent Person separation index, equal to 0.95), no evidence of bias due to item-trait interaction, high sensitivity and specificity, and control of major response sets [25]. In our study, Cronbach's alpha was 0.92. Pearson *r* for the correlation between the TDI and the BDI-II was 0.65 (*P* < 0.01).

### 2.3. Statistical Analysis

We used Velicer's Minimum Average Partial (MAP) test [40, 41] and structural equation modeling (SEM) to determine the factor model which best fitted the data.

The Velicer's MAP test has been proposed as a rule to find the best solution in exploratory factor analysis. It is accurate under many conditions, although, under certain conditions, it may have a tendency to underestimate the number of factors [42]. SEM was performed with a Robust Diagonally Weighted Least Squares Estimator (DWLSE) on a polychoric correlational matrix using the statistical package Lisrel 8.8 [43]. We evaluated the fit of the model by means of the following indexes: (a) the Root Mean Square Error of Approximation (RMSEA) [44, 45]; (b) the Incremental Comparative Fit Index (CFI) [45]; (c) the Satorra-Bentler scaled chi-square (SB *χ* ^2^); and (d) the Standardized Root Mean Square Residual (SRMSR) [45]. In the design and planning phase, we set a target recruitment size of 800 participants, greater than the standard of 10 participants per parameter estimated and a minimum of 300 participants reported in the literature [46, 47]. This sample size was set to have adequate power to have stability of the factorial solution.

According to the results from the SEM and the Velicer's MAP test, we performed an exploratory factor analysis using ordinary least squares to find minimum residual (MINRES). We used an oblique rotation method (ProMAX) because we expected that the ATS-R factors are correlated.

We reported Cronbach alphas, interitem mean correlations, and corrected item-total indices (i.e., discrimination indices) for the best-fitting factor model. Also, the convergent validity of the ATS-R with the BDI-II and the BHS was investigated by means of Pearson correlation coefficients. To test whether scores on the ATS-R dimensions were independently associated with depressive severity, we performed a generalized linear model with the BDI-II as dependent variable and the ATS-R factors and the BHS as independent variables. We reported odds ratio and their 95% confidence interval (CI) as indices of association. In addition, multivariate general linear model (GLM) was used to assess whether scores on the ATS-R factors were associated with the respondent's sex.

## 3. Results

### 3.1. Depression and Hopelessness Severity

One hundred twelve respondents (13.1% of the sample) had scores of 20 and higher on the BDI-II, indicative of moderate to severe depression, and 136 respondents (15.9% of the sample) had scores between 14 and 19, indicative of mild depression. Finally, 29.7% of the respondents reported scores of 9 or higher on the BHS, indicating severe hopelessness.

### 3.2. Factorial Validity

The Velicer's MAP test indicated that the best number of factors to extract was two. Furthermore, the three factor model proposed by Carver et al. [20] had poor fit (SB *χ*_32_^2^ = 321.04; *P* < 0.01; RMSEA = 0.10; 90% CI for RMSEA = 0.093/0.11; CFI = 0.94; SRMR = 0.075). 

As a result, we performed a MINRES factor analysis which indicated that items no. 1, no. 3, no. 4, no. 6, and no. 7 load on the first factor (“High Standards/Self-Criticism”), while items no. 2, no. 8, no. 9, and no. 10 load on the second factor (“Negative Generalization”) (see Table 1). The item no. 5, which originally was included in the Negative Generalization factor, did not load on any factor, while items no. 3, no. 6, and no. 9, which originally were included in the Self-Criticism factor, loaded sparsely on the “High Standards/Self-Criticism” (items no. 3, no. 6) and Negative Generalization (item no. 9) factors.

### 3.3. Reliability and Convergent Validity

Factors had satisfactory homogeneity of content (High Standards/Self-Criticism: Cronbach *α* = 0.81; mean interitem correlation = 0.46. Negative Generalization: Cronbach *α* = 0.75; mean interitem correlation = 0.43) (see Table 1) and correlated significantly with the BHS (High Standards/Self-Criticism: Pearson *r* = −0.29; *P* < 0.01. Negative Generalization: Pearson *r* = 0.22; *P* < 0.01). The pattern of correlations with measures of depression was less clear: Negative Generalization correlated significantly with the BDI-II (Pearson *r* = 0.29; *P* < 0.01), and nonsignificantly with the TDI (Negative Generalization: Pearson *r* = 0.01; *P* = 0.99), while High Standards/Self-Criticism correlated significantly with the TDI (Pearson *r* = −0.26; *P* < 0.01) and nonsignificantly with the BDI-II (Pearson *r* = 0.01; *P* = 0.67). 

The GLM indicated that both the ATS-R factors were independently associated with the BDI-II even when controlling for the severity of hopelessness. Young adults with higher depressive symptoms severity were (1) 1.23 times more likely to have higher High Standards/Self-Criticism scores (95% confidence interval: 1.08/1.39; *P* < 0.01); (2) 1.36 times more likely to have higher Negative Generalization scores (95% confidence interval: 1.17/1.58; *P* < 0.001); and (3) 2.66 times more likely to have higher BHS scores (95% confidence interval: 2.28/3.11; *P* < 0.001).

The GLM analysis indicated that sex had a significant effect (Wilks *λ* = 0.97; *P* < 0.001): men and women differed for High Standards/Self-Criticism mean scores (17.78 ± 3.99 and 16.38 ± 5.02, resp., for males and females; *F* = 18.08; *P* < 0.001) but not for Negative Generalization scores (11.29 ± 3.80 and 11.78 ± 4.09, resp., for males and females; *F* = 3.01; *P* = 0.08).

## 4. Discussion

Our results indicated that the three-factor solution obtained by Carver et al. [20] did not fit well the structure of the Italian version of the ATS-R. In our sample of Italian young adults, the ATS-R seems to measure two self-regulatory attitudes toward the self: the tendency to generalize from a single failure to a broader sense of personal inadequacy (“Negative Generalization”) and one dimension assessing the tendency to hold overly high standards and to be self-critical (“High Standards/Self-Criticism”). Furthermore, the pattern of loadings was partly discordant from that reported by Carver et al. [20]. Nonetheless, the pattern of loadings resulting from the factor analysis is consistent with the results reported by Carver [21]: in their research, the authors reported that Self-Criticism was significantly associated with the dimensions High Standards (*r* = 0.33; *P* < 0.01) and Negative Generalization (*r* = 0.52; *P* < 0.01), while the High Standards factor was almost unrelated to Negative Generalization (*r* = 0.05; *P* = n.s.) factor. In fact, in our sample, Negative Generalization and High Standards resulted in two independent factors, while the items originally loading on Self-Criticism loaded sparsely on the two remaining factors.

Our findings about the convergent validity of the ATS-R are only partially consistent with those reported by Carver et al. [20] and Carver [21]. While in the bivariate analyses, only the dimension of Negative Generalization correlated significantly with depression as measured with the BDI-II; at the multivariate level also the dimension High Standards/Self-Criticism resulted independently associated with depression, and not only the BHS and the Negative Generalization factor. These results suggest that a tendency to generalize from a single failure to a broader sense of personal inadequacy, a pessimistic view of the future, the holding of overly high standards, and the tendency to be self-critical are all important in the phenomenology of depression. However, there could be some differences between sexes. For example, while male and female Italian young adults did not differ in their tendency to generalize from a single failure to a broader sense of personal inadequacy, male young adults reported to hold more overly high standards and the tendency to be more self-critical at any failure to perform well. Furthermore, the effect sizes of the correlations between Negative Generalization, hopelessness, and depression were stronger in men than in women. This is discordant with Carver [21], who investigated the role of the ATS-R dimensions as prospective predictors of depressive symptoms in 336 US undergraduates. In their sample, the author reported that women had higher tendency toward Negative Generalization than did men, but the groups did not differ for the tendency to be self-critical or the holding of overly high standards. However, the author did not investigate whether the size of the associations between the ATS-R dimensions and depression may differ between sexes.

Reliability indices for the ATS-R factors were satisfactory but not good (Cronbach alphas between 0.75 and 0.81), due to a good homogeneity of content (mean interitem correlation >0.40 for both the factors), but the low number of items included in each factor. For Carver et al. [20], the brevity of the scale is a strength of the ATS-R, reflecting the fact that the essence of the cognitive tendencies measured with the ATS-R is “relatively straightforward.” However, from a psychometric point of view, scale's brevity might be a limitation for the use of the instrument in clinical settings [48].

Our findings have some limitations in their generalizability. First, we examined cognitive diatheses for depression in a sample of young adults recruited from the general population, a population in which the level of depression is generally low. Second, we used only self-report measures potentially biased by social desirability. Third, we did not assess other important psychometric characteristics of the ATS-R, such as the test-retest reliability. On the other hand, to our knowledge, this is the first study examining psychometric properties of the ATS-R in a sample of Italian young adults.

In conclusion, the Italian version of the ATS-R seems to be a valid instrument for the study of the role of cognitive tendencies as potential diathesis in the development of depression. Further research is needed to verify whether the psychometric properties and the factor structure of the ATS-R obtained in our sample are also replicable in clinical samples and in samples of adolescents and older adults.

## Figures and Tables

**Table 1:** Factor loadings and reliability indices.

	Factor loadings	Corrected item-total correlation	Cronbach's alpha if item deleted
High Standards/Self-Criticism			
ATS-R no. 1	0.77	0.62	0.77
ATS-R no. 3	0.61	0.63	0.77
ATS-R no. 4	0.76	0.56	0.79
ATS-R no. 6	0.62	0.64	0.76
ATS-R no. 7	0.69	0.54	0.79
Cronbach *α* = 0.81; mean interitem correlation = 0.46			
Negative Generalization			
ATS-R no. 2	0.77	0.58	0.68
ATS-R no. 8	0.61	0.51	0.71
ATS-R no. 9	0.52	0.46	0.74
ATS-R no. 10	0.85	0.64	0.64
Cronbach *α* = 0.75; mean interitem correlation = 0.43			
